# Enhancement of CCL2 expression and monocyte migration by CCN1 in osteoblasts through inhibiting miR-518a-5p: implication of rheumatoid arthritis therapy

**DOI:** 10.1038/s41598-017-00513-0

**Published:** 2017-03-24

**Authors:** Cheng-Yu Chen, Lih-Jyh Fuh, Chien-Chung Huang, Chin-Jung Hsu, Chen-Ming Su, Shan-Chi Liu, Yu-Min Lin, Chih-Hsin Tang

**Affiliations:** 1 Graduate Institute of Basic Medical Science, China Medical University, Taichung, Taiwan; 2 Department of Prosthodontics, China Medical University Hospital, Taichung, Taiwan; 3 Division of Immunology and Rheumatology, Department of Internal Medicine, China Medical University Hospital, Taichung, Taiwan; 4 School of Chinese Medicine, China Medical University, Taichung, Taiwan; 5 Department of Orthopedic Surgery, China Medical University Hospital, Taichung, Taiwan; 6 Department of Biomedical Sciences Laboratory, Affiliated Dongyang Hospital of Wenzhou Medical University, Dongyang, Zhejiang, China; 7 Institute of Medicine, Chung Shan Medical University, Taichung, Taiwan; 8 Department of Orthopedic Surgery, Taichung Veterans General Hospital, Taichung, Taiwan; 9 Department of Pharmacology, School of Medicine, China Medical University, Taichung, Taiwan; 10 Department of Biotechnology, College of Health Science, Asia University, Taichung, Taiwan

## Abstract

Cysteine-rich 61 (Cyr61 or CCN1), a secreted protein from the CCN family, is an important proinflammatory cytokine. Migration and infiltration of mononuclear cells to inflammatory sites play a critical role in the pathogenesis of rheumatoid arthritis (RA). Monocyte chemoattractant protein-1 (MCP-1/CCL2) is the key chemokine that regulates migration and infiltration of monocytes. Here, we examined the role of CCN1 in monocyte migration, and CCL2 expression in osteoblasts. We found higher levels of CCN1 and CCL2 in synovial fluid from RA patients compared with levels from non-RA controls. We also found that the CCN1-induced increase in CCL2 expression is mediated by the MAPK signaling pathway and that miR-518a-5p expression was negatively regulated by CCN1 via the MAPK cascade. In contrast, inhibition of CCN1 expression with lentiviral vectors expressing short hairpin RNA ameliorated articular swelling, cartilage erosion, and infiltration of monocytes in the ankle joints of mice with collagen-induced arthritis. Our study describes how CCN1 promotes monocyte migration by upregulating CCL2 expression in osteoblasts in RA disease. CCN1 could serve as a potential target for RA treatment.

## Introduction

Rheumatoid arthritis (RA) is a systemic autoimmune inflammatory disorder, in which the immune system attacks the healthy lining of joints^1, 2^. Pathological features of RA include inflammation, synovial swelling, pannus formation, stiffness in the joints and articular cartilage destruction^3, 4^. Most RA research has focused on cartilage destruction and synovial membrane inflammation. Less research has examined the involvement of subchondral bone erosions in RA pathogenesis^5–7^. Recent reports have indicated that subchondral bone plays a crucial role in cartilage pathology during the progression of RA^8, 9^. Therefore, better treatment strategies will ultimately require an improved understanding of the molecular mechanisms in subchondral bone in RA pathogenesis.

Accumulating evidence indicates that mononuclear cell migration plays an important step in the perpetuation of inflammation in RA^10^. Monocyte chemoattractant protein-1 (MCP-1), also known as chemokine ligand 2 (CCL2), belongs to the CC chemokine family. CCL2 is one of the key factors involved in the initiation of inflammation. It triggers chemotaxis and transendothelial migration of monocytes to inflammatory lesions^11^. CCL2 has been implicated in the pathophysiology of several inflammatory conditions including atherosclerosis, multiple sclerosis, arthritis and various cancers, via the attraction of monocytes and lymphocytes to the site of inflammation^12^. In addition, CCL2 levels are increased in the blood, synovial fluid, and synovial tissue of patients with RA compared with non-RA controls^13, 14^. Injection of CCL2 into rabbit joints resulted in marked macrophage infiltration in the affected joint^14^ and other preclinical evidence indicates that overexpression of CCL2 induces synovitis^15^, suggesting that CCL2 is a key regulator in monocyte migration and the inflammatory response during RA pathogenesis.

Cysteine-rich protein 61 (Cyr61/CCN1) is a component of the extracellular matrix and plays a role in endothelial cell adhesion, migration, proliferation, and differentiation^16^. Recently, it has been discovered that CCN1 promotes inflammation and regulates the effects of cytokines in chronic disease^17^. Moreover, CCN1 was necessary for interleukin-17 (IL)-17-induced fibroblast-like synoviocytes proliferation^18^ and promotes proinflammatory cytokine IL-1β production during the progression of RA disease^19^, and enhances VEGF-A-dependent angiogenesis in RA process^20^. These reports suggest that CCN1 plays a critical role in the pathogenesis of RA.

CCL2 is a chief regulator of monocyte infiltration in RA, which is constitutively expressed in the subchondral bone and may be associated with osteoblasts^21^. Although it is recognized that CCN1 is involved in RA pathogenesis, the role of CCN1 in CCL2 expression and monocyte accumulation is unclear. Our study results show that CCN1 induces upregulation of CCL2 expression in osteoblasts and subsequently promotes monocyte migration. In addition, *in vivo* results from our study show that lentiviral knockdown of CCN1 significantly abolishes bone erosion and monocyte infiltration in joints of mice with collagen-induced arthritis (CIA). These results provide new insights into the mechanisms of CCN1 action that may have therapeutic implications for patients with RA.

## Results

### High levels of CCN1 and CCL2 expression in RA synovial fluid

**Figure 1:**
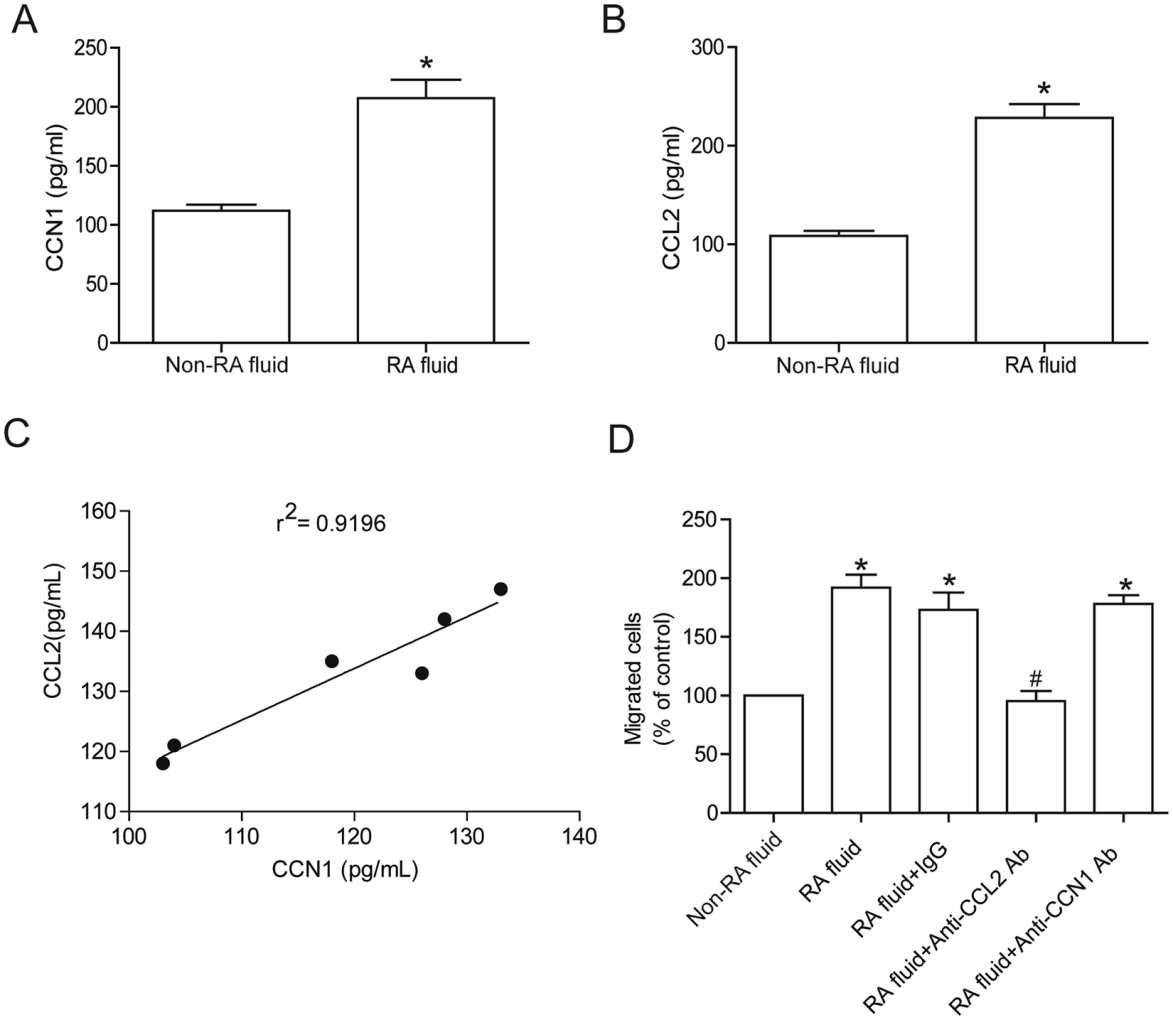
CCN1 and CCL2 are highly expressed in synovial fluid from patients with RA. (**A** and **B**) Synovial fluid from 6 patients with RA and 4 patients with non-RA was subjected to ELISA testing to determine levels of CCN1 and CCL2. (**C**) Correlation between CCN1 and CCL2 protein expression in synovial fluid from RA patients. (**D**) Human RA or non-RA fluid was pretreated with goat anti-CCL2, anti-CCN1 or goat IgG for 30 min, monocyte (THP-1) migration was assessed by *in vitro* chemotaxis assay (n = 4 per group). Results are expressed as the mean ± S.E. *p < 0.05 compared with non-RA patients.

CCN1 promotes the production of inflammatory cytokines such as IL-6 and oncostatin M (OSM) during the RA disease process^17, 22^. To characterize the role of CCN1 in CCL2 expression and monocyte infiltration during RA, we first analyzed CCN1 and CCL2 expression profiles in RA patients. We found that levels of CCN1 and CCL2 were significantly higher in synovial fluid from patients with RA compared with those from non-RA patients (Fig. 1A and B). The quantitative data also showed a highly positive correlation between the expression of CCN1 and CCL2 in RA patients (Fig. 1C). These results indicate that CCN1 and CCL2 levels are elevated in RA synovial fluid. We then examined whether CCN1 and CCL2 in RA synovial fluid enhance monocyte infiltration. An *in vitro* chemotaxis assay revealed monocyte migration in response to RA synovial fluid, which was attenuated by pre-incubation for 30 min with an anti-CCL2 neutralizing antibody; no such effect was seen with anti-CCN1 neutralizing antibody (Fig. 1D), implying that CCL2 is an important factor in monocyte infiltration during RA. Next, we examined whether CCN1 regulates CCL2-mediated monocyte migration in RA disease.

### CCN1 promotes CCL2 expression and enhances monocyte migration in osteoblasts

**Figure 2:**
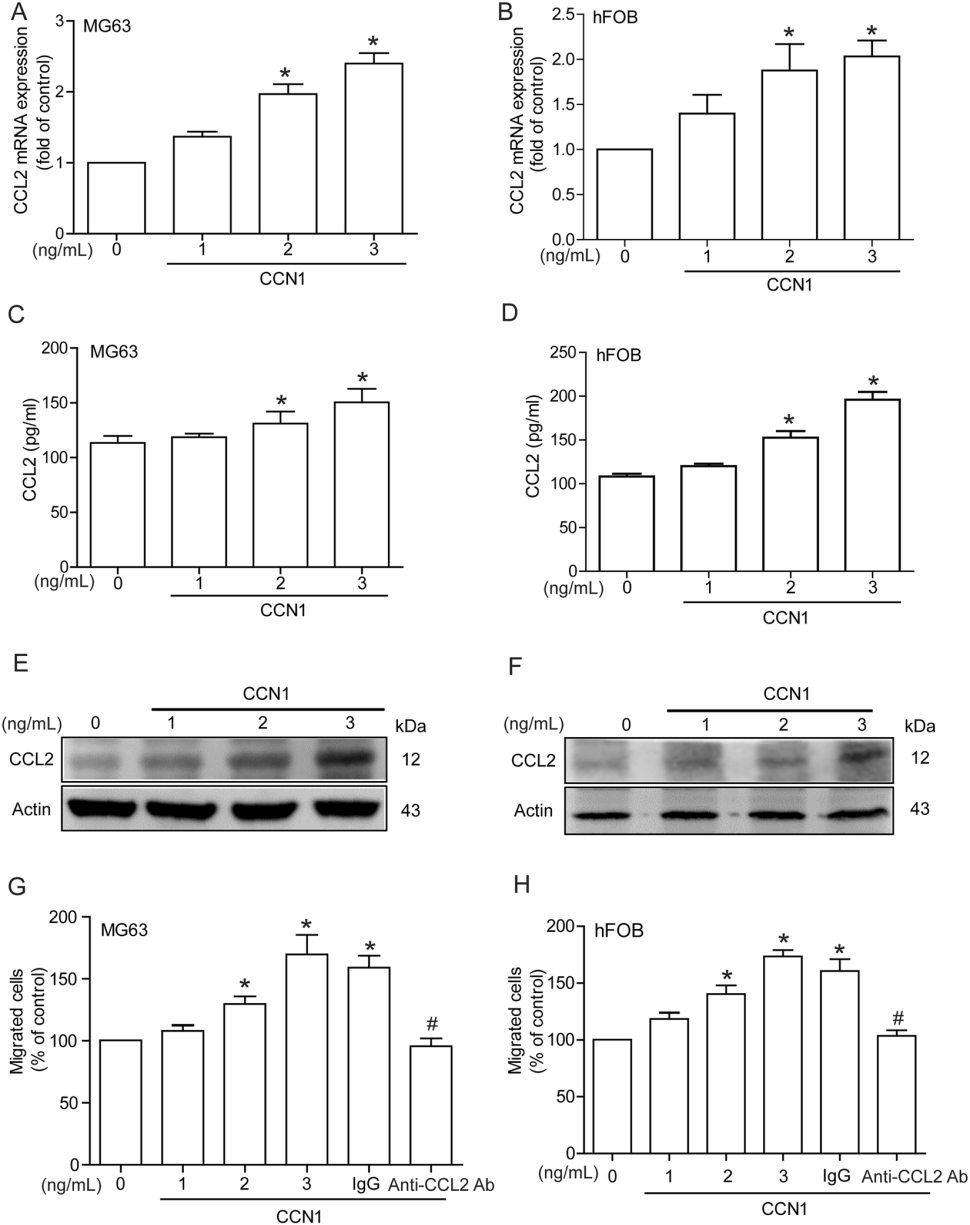
CCN1 promotes CCL2-dependent monocyte migration. (**A**–**F**) MG63 and hFOB cells were incubated with various concentrations of CCN1 (0–3 ng/ml) for 24 h; CCL2 expression was examined by qPCR, ELISA, and Western blot analysis (n = 5 per group). (**G** and **H**) Osteoblasts were incubated with CCN1 (0–3 ng/ml) for 24 h, or pretreated for 30 min with IgG control antibody or CCL2 antibody, followed by stimulation with CCN1 (3 ng/ml) for 24 h. Medium was collected as CM and THP-1 migration was assessed by *in vitro* chemotaxis assay (n = 5 per group). Results are expressed as the mean ± S.E. *p < 0.05 compared with controls.

Emerging evidence indicates that subchondral bone plays an essential role in the mechanisms of bone remodeling during the development of RA^23^. We examined CCL2 production in osteoblasts after CCN1 treatment. Treatment of osteoblasts (MG63 and hFOB cells) increased CCL2 mRNA expression and protein secretion in a concentration-dependent manner (Fig. 2A–F). In addition, conditioned medium (CM) from CCN1-treated osteoblasts dramatically enhanced monocyte migration and this effect was blocked by a CCL2 mAb (Fig. 2G–J), which implies that CCN1 promotes monocyte migration through a CCL2-dependent pathway. Integrin αvβ3 has been implicated in CCN1-induced OSM expression in osteoblasts^17^. We found that treatment of osteoblasts with αvβ3 integrin mAb abolished CCN1-induced CCL2 expression (Supplementary Fig. S1), demonstrating that integrin αvβ3 is involved in CCN1-induced promotion of CCL2 expression.

### CCN1 promotes CCL2 expression and monocyte migration through the MAPK pathway

**Figure 3:**
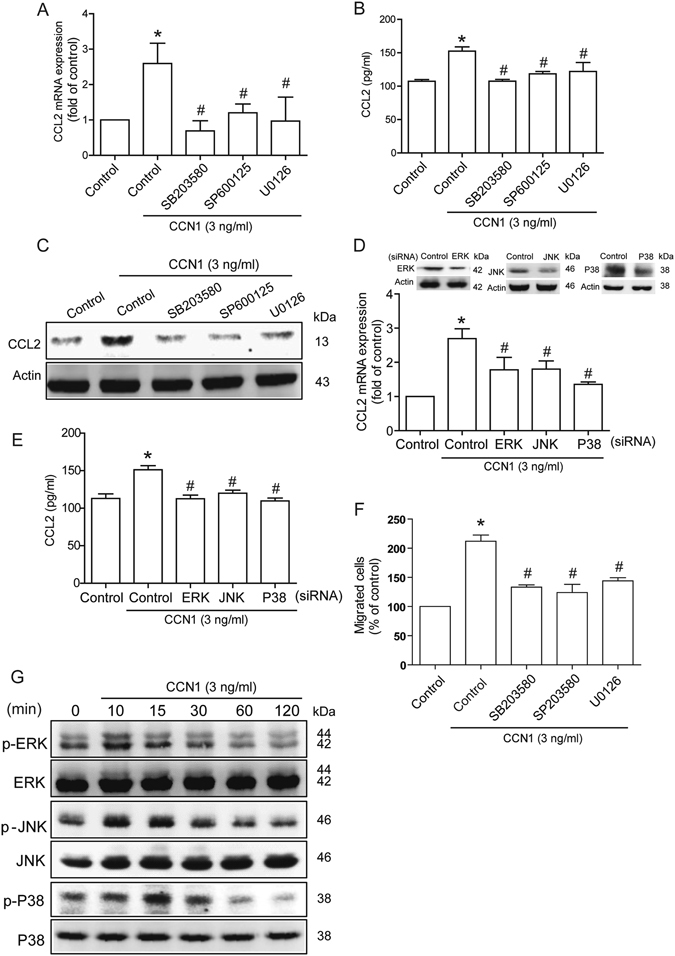
The MAPK signaling pathway is involved in CCN1-induced CCL2 expression. (**A**–**E**) MG63 cells were pretreated with SB203580 (10 μM), SP600125 (3 μM), or U0120 (10 μM) for 30 min, or transfected with ERK, JNK, and p38 siRNA for 24 h, followed by stimulation with CCN1 for 24 h. CCL2 expression was examined by qPCR, ELISA and Western blot analysis (n = 5 per group). (**F**) Medium was collected as CM, and THP-1 migration was assessed by *in vitro* chemotaxis assay (n = 5 per group). (**G**) MG63 cells were incubated with CCN1 (3 ng/ml) for indicated time intervals, and ERK, JNK, and p38 phosphorylation was examined by Western blot analysis (n = 4). Results are expressed as the mean ± S.E. *p < 0.05 compared with controls. ^#^ *p* < 0.05 compared with CCN1-treated group.

Mitogen-activated protein kinases (MAPKs) ERK, JNK, and p38 MAPK are involved in the regulation of CCL2 expression^24, 25^. We therefore investigated the role of MAPKs in mediating CCN1-induced CCL2 expression, using the specific p38 inhibitor SB203580, JNK inhibitor SP600125, and MEK1/2 inhibitor U0126. Pretreatment of osteoblasts with these agents reversed CCN1-induced CCL2 expression (Fig. 3A–C). In addition, osteoblast transfection with ERK, JNK and p38 siRNA markedly inhibited CCN1-enhanced CCL2 production (Fig. 3D and E). CCN1-mediated monocyte migration was also reduced by pretreatment with SB203580, SP600125, and U0126 (Fig. 3F). Moreover, incubation of osteoblasts with CCN1 promoted ERK, JNK and p38 phosphorylation in a time-dependent manner (Fig. 3G). Thus, CCN1 appears to act through the MAPK signaling pathway to promote CCL2 expression in osteoblasts and subsequently enhance monocyte migration.

### CCN1 promotes CCL2 expression via inhibition of miR-518a-5p expression

**Figure 4:**
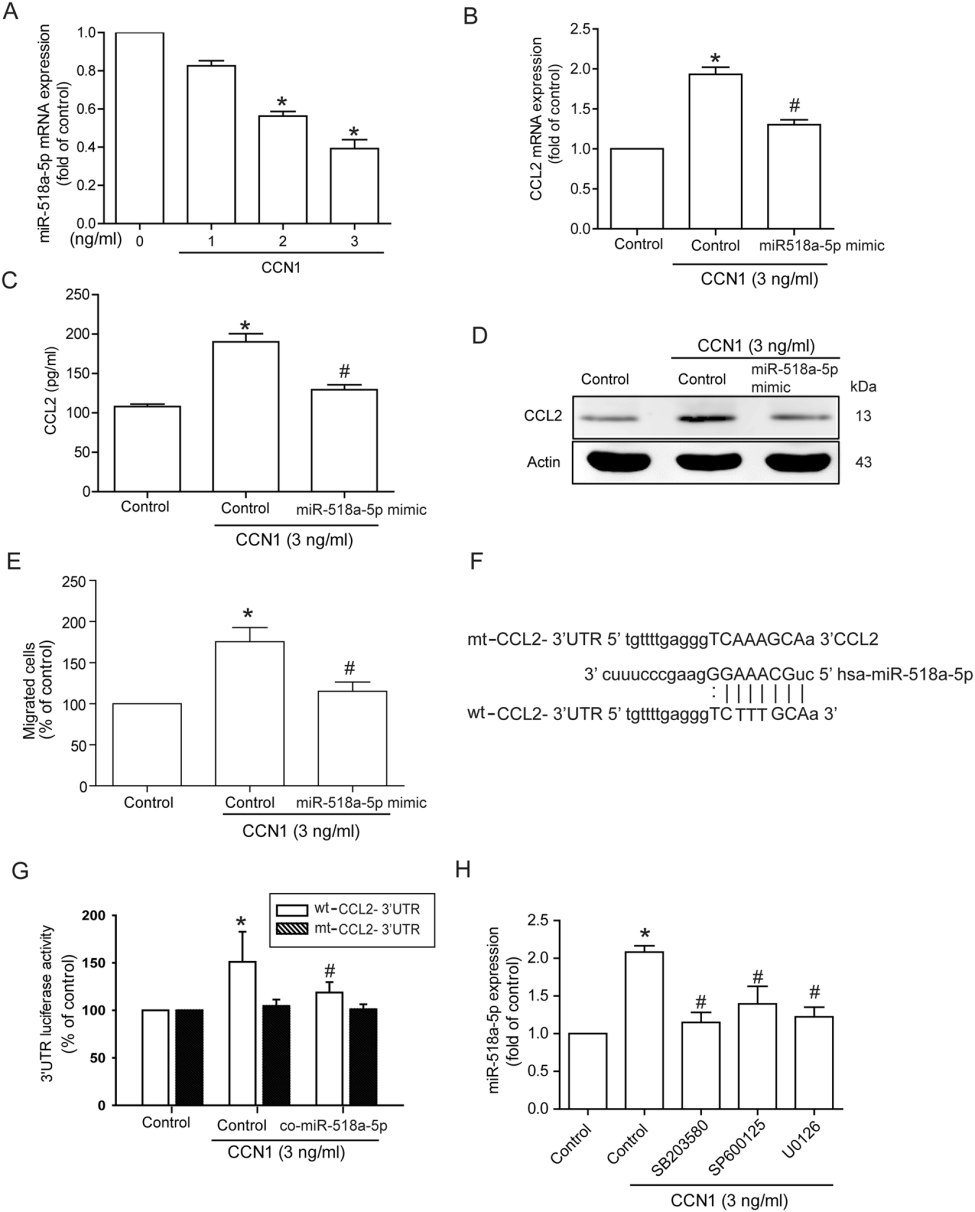
CCN1 promotes CCL2 expression and monocyte migration via inhibition of miR-518a-5p. (**A**) MG63 cells were incubated with CCN1 (1–3 ng/ml) for 24 h; miR-518a-5p expression was examined by qPCR (n = 5 per group). (**B**–**D**) MG63 cells were transfected with control miRNA or miR-518a-5p mimic for 24 h followed by stimulation with CCN1 for 24 h, and CCL2 expression was examined by qPCR, ELISA and Western blot analysis (n = 5 per group). (**E**) Medium was collected as CM and THP-1 migration was assessed by *in vitro* chemotaxis assay (n = 5 per group). (**F**) Schematic 3′UTR representation of the human CCL2 containing the miR-518a-5p binding site. (**G**) MG63 cells were transfected with wt-CCL2-3′UTR or mt-CCL2-3′UTR plasmid for 24 h and stimulated with CCN1 for 24 h; relative luciferase/renilla activities were measured as described in the Methods section (n = 4 per group). (**H**) MG63 cells were pretreated with SB203580, SP600125, and U0126 for 30 min followed by stimulation with CCN1 for 24 h, and miR-518a-5p expression was examined by qPCR (n = 5 per group). Results are expressed as the mean ± S.E. *p < 0.05 compared with controls. ^#^ *p* < 0.05 compared with CCN1-treated group.

miRNAs are important regulators in RA, which makes them promising therapeutic targets^26^. Open-source software (TargetScan, miRDB, and PicTar) was used to predict and identify target miRNAs. These tools revealed that the 3′UTR region of CCL2 mRNA harbors potential binding sites for miR-518a-5p. Exogenous CCN1 reduced miR-518a-5p expression in a concentration-dependent manner (Fig. 4A). We found that transfection of osteoblasts with the miR-518a-5p mimic reduced CCN1-induced CCL2 expression (Fig. 4B–D) and reduced CCN1-promoted monocyte migration (Fig. 4E). Thus, CCN1 promotes CCL2 expression and monocyte migration by suppressing miR-518a-5p expression.

To learn whether miR-518a-5p regulates the 3′UTR region of CCL2, we constructed a luciferase reporter vector harboring the wild-type (wt-CCL2-3′UTR) or mutant (mt-CCL2-3′UTR) 3′UTR region of CCL2 mRNA (Fig. 4F). The results show that CCN1 increased wild-type but not mutant CCL2 3′UTR luciferase activity (Fig. 4G). Transfection with miR-518a-5p mimic inhibited CCN1-induced wild-type CCL2-3′UTR luciferase activity (Fig. 4G), while treatment with U0126, SB203580, and SP600125 reversed CCN1-inhibited miR-518a-5p expression (Fig. 4H). These data suggest that miR-518a-5p directly represses CCL2 protein expression via binding to the 3′UTR region of the human *CCL2* gene through MAPK signaling.

### Knockdown of CCN1 impairs CIA-induced RA and monocyte infiltration

**Figure 5:**
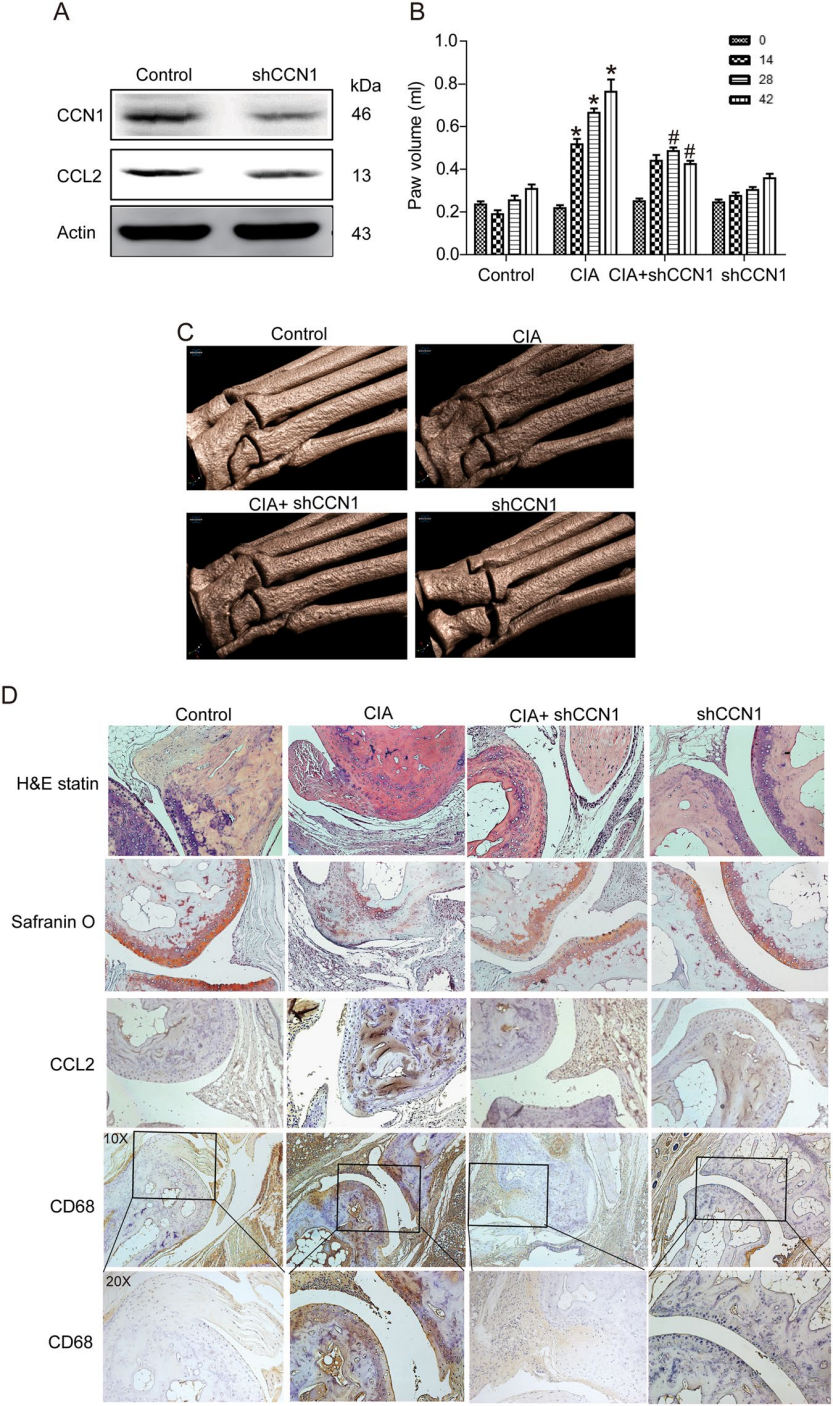
Lenti-shCCN1 attenuates bone erosion and monocyte migration in CIA mice. (**A**) CCN1 and CCL2 expression in control-shRNA MG63 and CCN1-shRNA MG63 cells was examined by Western blot analysis (n = 4). (**B**) The control and CIA mice were administered intra-articular injections of 7.1 × 10^6 ^PFU Lenti-shCCN1 on day 14 and sacrificed on day 42. Swelling of hind paws was measured by a digital plethysmometer (n = 5 per group). (**C**) Representative micro-CT images of the hind paws on day 42. (**D**) Histologic sections of ankle joints were stained with H&E or safranin O and immunostained with CCL2 or CD68; Scale bar, 50 *μ*m. Results are expressed as the mean ± S.E. *p < 0.05 compared with controls. #*p* < 0.05 compared with CCN1-treated group.

To confirm CCN1-mediated CCL2 expression and monocyte infiltration *in vivo*, CCN1-shRNA was transfected into osteoblasts, thus reducing CCN1 and CCL2 expression (Fig. 5A). Next, we used the CIA model to investigate whether the knockdown of CCN1 was able to inhibit arthritis *in vivo*. Within 4 weeks, mice had a very high incidence of arthritis, and 95% of animals developed severe arthritis. Mice injected with Lenti-shCCN1 had significantly attenuated paw swelling compared with the control group, as reflected by hind paw volume data (Fig. 5B). Micro-computed tomography (micro-CT) imaging of hind paws showed that bone erosion was significantly increased in the CIA mice (Fig. 5C), whereas Lenti-shCCN1-infected had lower levels of bone erosion compared with controls (Fig. 5C). Intra-articular cartilage erosion and proteoglycan content were assayed by staining with H&E and safranin O (Fig. 5D). IHC staining of the ankle joints revealed that the number of CD68- (monocytes/macrophages marker) and CCL2-positive cells were significantly increased in mice with CIA but markedly diminished in CIA mice treated with Lenti-shCCN1 (Fig. 5D). Our results confirmed that Lenti-shCCN1 prevents cartilage erosion and reduces monocyte infiltration *in vivo*.

## Discussion

RA is a systemic immune disease that causes chronic inflammation of the synovium and bone destruction in the joint. The inflammatory process is mediated through a complex cytokine and chemokine. It is not well understood as to which factors are responsible for initiating the degradation and loss of the articular tissues. CCL2 is believed to play an important role as a proinflammatory agent and assist in the accumulation of leucocytes and monocytes in RA synovial and fluid, increasing the intensity of synovial inflammation^27, 28^. CCN1 is involved in RA pathogenesis^29^; its effect on CCL2 expression and monocyte infiltration is largely unknown. CCN1 has been reported to promote CCL2 production in endothelial cells and endothelial progenitor cells^30^. This current study found significantly higher levels of CCN1 and CCL2 expression in RA synovial fluid compared with non-RA synovial fluid. Moreover, we identified CCL2 as a target protein for the CCN1 signaling pathway regulating monocyte infiltration. Treatment of osteoblasts with CCN1 increased CCL2 expression, inducing the migration of human monocytes. CM-mediated monocyte migration in osteoblasts was abolished by CCL2 mAb treatment. In addition, CCN1 knockdown reduced CIA-induced RA and monocyte infiltration. Our data suggest that CCN1 increases CCL2 expression and promotes monocyte migration in osteoblasts. This phenomenon may occur via inhibition of miR-518a-5p through the MAPK signaling pathway. We previous reported that CCN1 promotes VEGF-A expression in osteoblasts and subsequently enhances angiogenesis through the inhibition of miR-216 during RA disease^20^. Lin *et al.*, have also indicated that CCN1 induces proinflammatory cytokine IL-6 production in RA synovial fibroblasts^31^. Furthermore, CCN1 has been reported to induce other proinflammatory cytokines such as IL-1β and IL-17, as well as OSM expression during RA^17–19^. Our current results and previous findings suggest that CCN1 is a key regulator in RA and may serve as a therapeutic target in treatment using a small molecule inhibitor or CCN1 antibody.

CCN1 has been implicated in other autoimmune/inflammatory diseases besides RA. For instance, CCN1 can aggravate psoriasis^32^ and also enhance the pathogenesis of psoriasis through up-regulation of IL-8^33^. Conversely, other evidence indicates that CCN1 modulates the course of colitis^34^ through IL-6^35^. Furthermore, overexpression of CCN1 exacerbates obliterative bronchiolitis in mice^36^. Cigarette smoke extract-induced CCN1 augments IL-8, VEGF and MMP expression in bronchial epithelial cells^37, 38^. This current study indicated that CCN1 mediates arthritis pathogenesis and supports the previous evidence suggesting that CCN1 plays a critical role in autoimmune/inflammatory diseases.

Studies have increasingly focused on the impact of inflammation in osteoblasts, which exhibit important functions within the arthritic bone microenvironment and RA pathogenesis^39, 40^. These studies highlight that the influence of inflammation on bone is specific to the site of inflammation and dependent upon the cytokines present within the local bone microenvironment^39, 40^. CCL2 is constitutively expressed in the subchondral bone and associated with osteoblasts in RA^21^. Our results support these concepts and we found that CCN1 promotes osteoblast production of CCL2 and subsequently induces monocyte migration. Knockdown CCN1 inhibited CIA-induced RA and monocytes/macrophages marker expression in subchondral bone *in vivo*. Our results provide evidence that osteoblast-mediated monocyte migration plays a crucial role during the arthritis disease process. In this study, we did not perform shRNA-control in CIA mice. However, intra-articular injected shCCN1 did not affect the paw swelling, cartilage degradation and bone erosion in mice. In addition, previous studies indicated that Lenti-negative control shRNA didn’t have any effects when compared with the CIA group^41–43^. Therefore, the Lentivirus delivery effect can be rule out.

Evidence indicates that the MAPK signaling pathway plays an important role in the regulation of gene expression levels^44^. Here, we report that the MEK1/2 inhibitor U0126, p38 inhibitor SB203580, and JNK inhibitor SP600125 antagonized CCN1-induced CCL2 expression. Conversely, these inhibitors blocked CCN1-promoted monocyte migration. In this study, incubation of osteoblastic cells with CCN1 promoted ERK, p38, and JNK phosphorylation, suggesting that MAPK activation plays a key role in CCN1-induced CCL2 production and monocyte infiltration.

Several studies have focused on the role of miRNAs in gene regulation^45^. As miRNAs are able to modulate target gene translation via binding to the 3′-UTR of mRNAs and thus affect multiple protein-encoding genes at the post-transcriptional level, they are implicated in the control of a wide range of biological functions^46^. Therefore, we investigated whether miRNAs are involved in monocyte migration following CCN1 stimulation. The current study showed that CCN1 markedly represses miR-518a-5p expression in osteoblasts. Co-transfection of cells with miR-518a-5p mimic abolished CCN1-induced CCL2 expression and monocyte migration. Strikingly, we found that miR-518a-5p directly inhibited CCL2 protein expression through binding to the 3′UTR of the human *CCL2* gene, thereby negatively regulating CCL2-mediated monocyte migration. These findings provide insight into potential miRNA-based strategies for CCL2-mediated monocyte migration.

In conclusion, our study has identified that CCN1 promotes CCL2 expression in osteoblasts through the negative regulation of miR-518a-5p via the MAPK signaling pathway, leading to the depletion of monocyte infiltration. CCN1 may be a novel therapeutic target in RA.

## Materials and Methods

### Materials

Rabbit polyclonal antibodies specific for β-actin, CCL2, p-ERK, ERK, p-p38, p38, p-JNK, JNK, and CD68 were purchased from Santa Cruz Biotechnology (Santa Cruz, CA). Mouse monoclonal antibody specific for CCN1 was purchased from R&D Systems (Minneapolis, MN). Recombinant human CCN1 was purchased from PeproTech (Rocky Hill, NJ). Dulbecco’s Modified Eagle Medium (DMEM), fetal bovine serum (FBS), and all other cell culture reagents were purchased from Gibco-BRL Life Technologies (Grand Island, NY). The dual-luciferase reporter assay kit was purchased from Promega (Madison, WI, USA). Negative control miRNA and human miR-518a-5p mimic were purchased from GeneDireX (Las Vegas, NV). All chemicals were purchased from Sigma-Aldrich (St. Louis, MO).

### Cell culture

The human osteoblast-like cell line MG63 and conditionally immortalized human fetal osteoblastic cell line hFOB were purchased from American Type Culture Collection (Manassas, VA). MG63 cells were cultured in MEM supplemented with 10% FBS and antibiotics (100 U/ml of penicillin and 100 μg/ml of streptomycin) and were maintained at 37 °C in a humidified atmosphere of 5% CO_2_. hFOB cells were cultured in a 1:1 mixture of phenol-free DMEM/Ham’s F12 medium containing 10% FBS supplemented with antibiotics at 33.5 °C, the optimal temperature for rapid proliferation of the large T antigen from the Simian Virus (SV40).

THP-1, a human leukemia cell line of monocyte/macrophage lineage, was obtained from American Type Culture Collection (Manassas, VA, USA) and grown in RPMI-1640 medium with 10% FBS^47, 48^.

### Human synovial fluids

We obtained study approval from the local ethics committee and all participants gave written informed consent before study enrolment. Synovial fluid was obtained from 6 RA patients undergoing total knee arthroplasty; non-RA synovial fluid was obtained from 4 patients during arthroscopy for trauma/joint derangement. The China Medical University Hospital Institutional Review Board approved the protocol, and all methods were carried out in accordance with the guidelines and regulations promulgated by the Hospital’s Institutional Review Board.

### Quantification of mRNA and miRNA by real-time quantitative polymerase chain reaction amplification

Total RNA was extracted from osteoblasts using a TRIzol^®^ kit (MDBio, Taipei, Taiwan) and miRNAs were quantified using the Mir-X^TM^ miRNA First-Strand Synthesis Kit (Clontech Laboratories, Palo Alto, CA), as per the manufacturers’ protocols. RNA quality and total RNA samples were examined using the Nanovue^TM^ Spectrophotometer (GE Healthcare, WI). Complementary DNA was derived from 1 μg of total RNA using an M-MLV Reverse Transcriptase kit (Invitrogen, Carlsbad, CA), according to the manufacturer’s recommendations. Real-time quantitative polymerase chain reaction (qPCR) analysis was carried out with the KAPA SYBR^®^ FAST qPCR Kit (Applied Biosystems, Foster City, CA). Relative normalization of gene expression was performed using endogenous GAPDH as the internal control for mRNA or small nuclear RNA U6 as the internal control for micro RNA (miRNA).

### Western blot analysis

Cellular lysates were prepared as according to our previous instructions^49^. Proteins were resolved under SDS-PAGE conditions, then transferred electrophorectically onto polyvinyldifluoride (PVDF) membranes (Immobilon, Bedford, MA). Blots were blocked with 4% BSA for 1 h at room temperature, then probed with primary antibodies against CCL2, p-ERK, ERK, p-p38, p38, p-JNK, JNK (1:1000) and β-actin (1:2000) for another hour at room temperature. After three washes, blots were subsequently incubated with the horseradish peroxidase-conjugated secondary antibodies for 1 h at room temperature and visualized by enhanced chemiluminescence using ImageQuant™ LAS 4000 (GE Healthcare, Pewaukee, WI).

### Enzyme-linked immunosorbent assay (ELISA)

Osteoblasts were cultured in 24-well plates until they reached 90% confluence and were then changed to serum-free medium, in which they were treated with CCN1 alone for 24 h or pretreated with pharmacological inhibitors, followed by 24 h of CCN1 stimulation. CM was collected, centrifuged and stored at −80 °C. CCL2 levels were assessed in the culture media using the CCL2 ELISA kit (R&D Systems; Minneapolis, MN), as per the manufacturer’s protocol.

### *In vitro* chemotaxis assay

Synovial fluid or CM obtained from cells treated with CCN1 or pharmacological inhibitors were placed in the lower compartment of Transwell cluster plates (Costa Corning, Cambridge, MA, USA); the two-compartment chambers were separated by a polycarbonate membrane filter. THP-1 cells (5 × 10^4^ cells) were added to the upper chamber and incubated at 37 °C for 4 h, then fixed in 3.7% formaldehyde for 5 min and stained with 0.05% crystal violet in phosphate-buffered saline (PBS) for 15 min. Cells on the upper side of the filters were removed with cotton-tipped swabs and the filters were washed with PBS. Cells on the underside of the filters were examined and counted under a microscope. A goat anti-mouse CCL2 or goat IgG was used in the chemotaxis assay media to determine the biological specificity of the above chemokines produced by the cells to stimulate monocyte migration.

### Plasmid construction

The 3′-untranslated region (3′UTR) of human CCL2 contains a miR-518a-5p binding site. A DNA fragment containing wt-CCL2 3′UTR (CCL2 3′-UTR) and mt-CCL2 3′UTR were purchased from Invitrogen. The fragment from 84~277 of CCL2 3′-UTR was subcloned into the luciferase reporter vector pmirGLO-control (Promega, Madison, WI), upstream of the vector’s promoter. The construct was confirmed by sequencing using the Applied Biosystems 3730xl DNA Analyzer (Thermo Fisher Scientific, NY).

### CCN1 knockdown in osteoblast cell lines

Recombinant lentiviruses were produced by transient cotransfection of 293 T cells with short hairpin (sh)RNA-expressing plasmid (TRCN0000118099) along with the packaging plasmid pCMVΔR8.91 and the VSV-G envelope glycoprotein expression plasmid pMD.G, all of which were obtained from the National RNAi Core Facility at the Academia Sinica in Taiwan. After 48 h, lentiviral particles carrying CCN1 shRNA (shCCN1) were isolated form the supernatant of 293 T cells. A plaque assay using serial dilution was performed in human osteoblasts to determine virus titers (in plaque-forming units [PFU]) of shCCN1. The viral titer of shCCN1 was determined to be ∼7.1 × 10^6^ PFU.

Osteoblasts were seeded in 6 cm plates for 24 h. Growth media was removed and replaced with fresh media containing polybrene (5 μg/ml). Cells were infected by addition of shCCN1 particles to the culture. After incubation overnight, the media was removed and replaced with 5 ml of fresh growth media containing 2 μg/ml puromycin, to select for stable transfectants. Single clones were picked, and the ectopic expression of the gene of interest was verified using Western blot analysis.

### Collagen-induced arthritis mouse model

All animal procedures were approved and performed in accordance with the guidelines of the Institutional Animal Care and Use Committee of China Medical University. 20 Male C57BL/6 J mice (aged 8–10 weeks, body weight 25 ± 3 g) were purchased from the National Laboratory Animal Centre (Taipei, Taiwan) and randomized to four groups: Control group, healthy mice; CIA group, CIA-induced arthritis; CIA + shCCN1 group, CIA-induced arthritis treated with shCCN1; shCCN1 group, healthy mice treated with shCCN1. The CIA mouse model protocol followed that detailed in previously published work^23,50^. First, 0.1 ml of an emulsion containing 100 mg of bovine type II collagen (CII) dissolved at a concentration of 2 mg/ml in 0.1 M acetic acid and complete Freund’s adjuvant was injected intradermally into the base of the tail. Two weeks after the primary immunization, a booster injection of 100 mg CII dissolved and emulsified 1:1 with incomplete Freund’s adjuvant was administered into the hind paw. The incidence of arthritis in CIA mice was very high within 6 weeks after the first immunization, and 95% of the mice developed severe arthritis.

The control and CIA mice were administered intra-articular injections of 7.1 × 10^6^ PFU Lenti-shCCN1 on day 14 and sacrificed on day 42. The severity of arthritis in each paw was measured in a blinded manner with a plethysmometer (Marsap, Mumbai, India) once weekly for 4 weeks. Upon sacrifice on day 42, phalanges and ankle joints were removed immediately and fixed in 4% paraformaldehyde for micro-CT analysis. For bone erosion analysis, the reconstructed images of tarsal bone and metatarsal bone were isolated. In brief, all individual samples were re-orientated to the same position and ankle using Dataviewer (SkyScan). 690 slices (2.0 mm) from the edge of tarsal bone were selected as the region of interest, as this area included tarsal bone and parts of metatarsal bone; CTAn software (SkyScan) was used to calculate the threshold value and morphometric indices of bone. For illustration, density rendering software CTVox (Version 3.0, SkyScan) was used to provide 3D images and all individual samples used the same color and lighting/shadow visualization.

### IHC (immunohistochemistry) staining

Paraffin-embedded sections were prepared, mounted on silane-coated slides, deparaffinized in xylene, rehydrated in a graded alcohol series and rinsed in deionized water. After antigen retrieval, intrinsic peroxidase activity was blocked by incubation with 3% H_2_O_2_. Non-specific antibody-binding sites were blocked using 3% BSA in PBS. Sections were then incubated with appropriately diluted primary antibodies specific for mouse CD68 or CCL2 at 4 °C overnight. After three washes inh PBS, the secondary antibody (biotin-labeled goat anti-rabbit IgG) was applied for 30 min at room temperature. Staining was detected with DAB (3,3′-diaminobenzidine tetrahydrochloride) and hematoxylin using a Novolink Polymer Detection Kit (Leica Biosystems). Slides were observed under a light microscope. Specimens from ankles of CIA mice were also stained with Safranin-O/Fast Green to evaluate cartilage lesions and hematoxylin and eosin (H&E) stain to investigate morphology.

### Statistical Analysis

All quantified results were calculated using GraphPad Prism 5.0 software and are presented as the mean ± standard error (S.E.) of at least three experiments. A statistical comparison of two groups was performed using the Student’s t test. Statistical comparisons of more than two groups were performed using the two-factor analysis of variance with the Bonferroni post hoc test or the Mann–Whitney U test, as appropriate. In all cases, *p* < 0.05 was considered statistically significant.

## Electronic supplementary material

Dataset 1
