# Supplementary material for: Enhancement of CCL2 expression and monocyte migration by CCN1 in osteoblasts through inhibiting miR-518a-5p: implication of rheumatoid arthritis therapy
Source: Sci Rep. 2017 Mar 24;7:421. doi: 10.1038/s41598-017-00513-0 (PMC5428676; doi:10.1038/s41598-017-00513-0)
Supplement: Supplementary file 1 — Dataset 1 [file 41598_2017_513_MOESM1_ESM.doc]

**Enhancement of CCL2 expression and monocyte migration by CCN1 in osteoblasts through inhibiting miR-518a-5p : implication of rheumatoid arthritis therapy**

Cheng-Yu Chen^1^, Lih-Jyh Fuh^2^, Chien-Chung Huang^3^, Chin-Jung Hsu^4,5^, Chen-Ming Su^6^, Shan-Chi Liu^1^, Yu-Min Lin^7,8*^ and Chih-Hsin Tang^1,9,10*^

^1^Graduate Institute of Basic Medical Science, China Medical University, Taichung, Taiwan

^2^Department of Prosthodontics, China Medical University Hospital, Taichung, Taiwan

^3^Division of Immunology and Rheumatology, Department of Internal Medicine, China Medical University Hospital, Taichung, Taiwan

^4^School of Chinese Medicine, China Medical University, Taichung, Taiwan

^5^Department of Orthopedic Surgery, China Medical University Hospital, Taichung, Taiwan

^6^Department of Biomedical Sciences Laboratory, Affiliated Dongyang Hospital of Wenzhou Medical University, Dongyang, Zhejiang, China

^7^Institute of Medicine, Chung Shan Medical University, Taichung, Taiwan

^8^Department of Orthopedic Surgery, Taichung Veterans General Hospital, Taichung, Taiwan

^9^Department of Pharmacology, School of Medicine, China Medical University, Taichung, Taiwan

^10^Department of Biotechnology, College of Health Science, Asia University, Taichung, Taiwan

***Corresponding author**

Chih-Hsin Tang; PhD

Graduate Institute of Basic Medical Science, China Medical University E-mail: [chtang@mail.cmu.edu.tw](mailto:wenmei@ha.mc.ntu.edu.tw)

Or

Yu-Min Lin; MD, PhD

E-mail: [ymlin@vghtc.gov.tw](mailto:wenmei@ha.mc.ntu.edu.tw)

**Supplementary data**


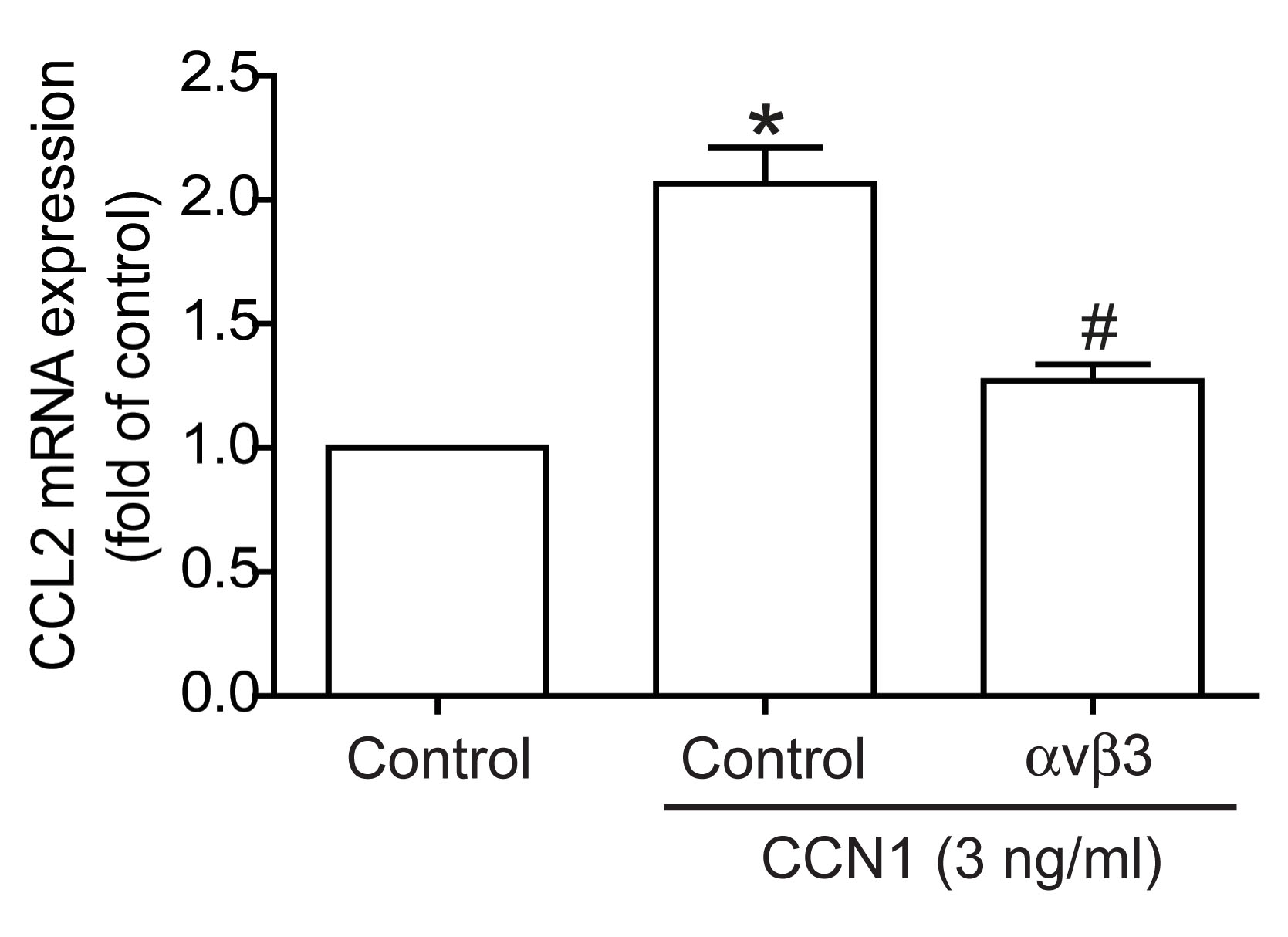


**Fig. S1. CCN1 increases CCL2 expression in through αvβ3 integrin.** MG63 cells were pretreated with αvβ3 integrin antibody for 30 min followed by stimulation with CCN1 for 24 h, the CCL2 expression was examined by qPCR. Results are expressed as the mean ± S.E. *, *p* < 0.05 compared with control. #, *p* < 0.05 compared with the CCN1-treated group.


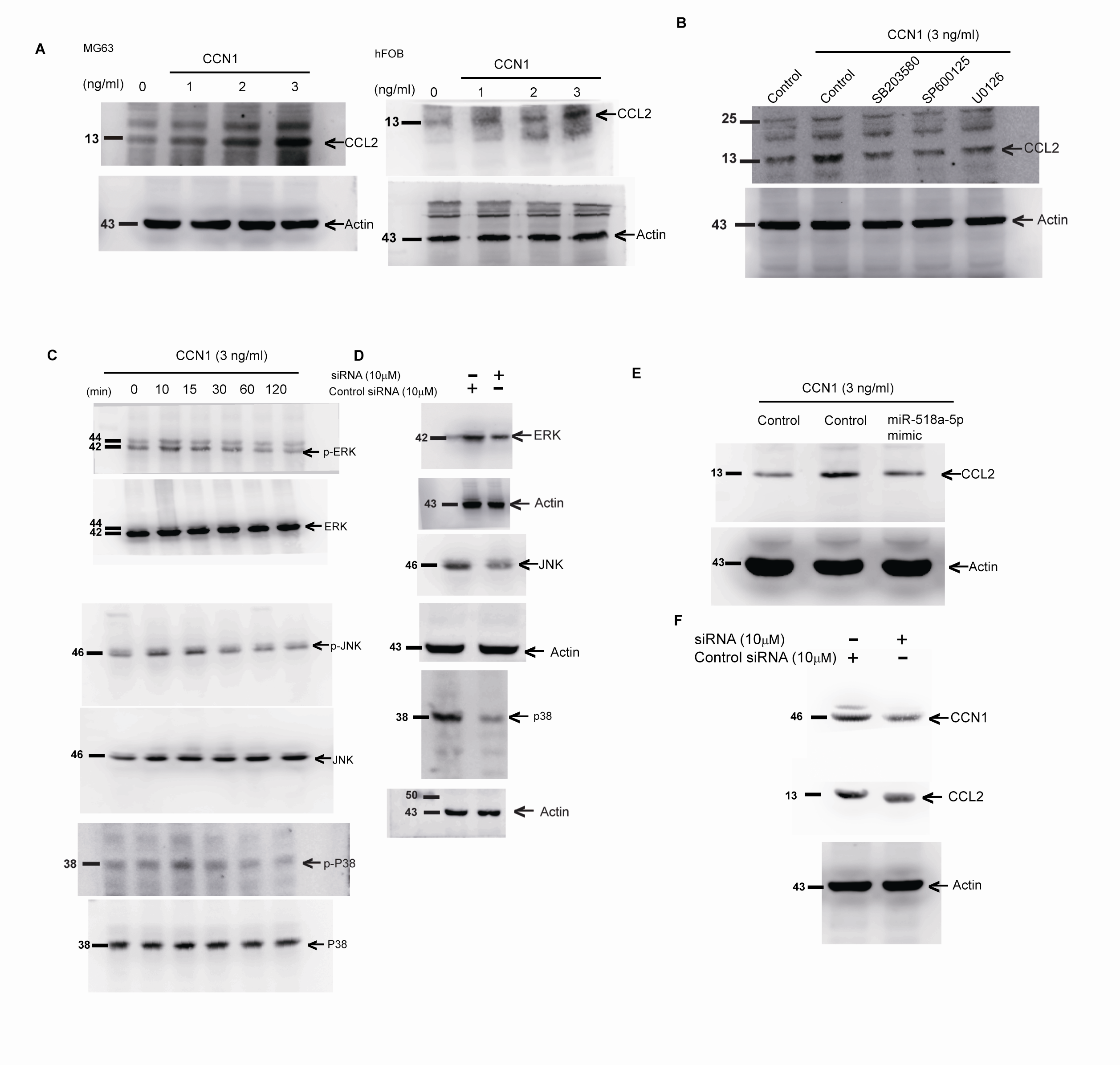


**Fig. S2. Full Western blots of Fig. 2-5.**

Fig. S2A: full Western blot of Fig.2E and F.

Fig. S2B: full Western blot of Fig.3C.

Fig. S2C: full Western blot of Fig.3G.

Fig. S2D: full Western blot of Fig.3D.

Fig. S2E: full Western blot of Fig.4D.

Fig. S2F: full Western blot of Fig.5A
